# The Restorative Potential of Icelandic Nature

**DOI:** 10.3390/ijerph17239095

**Published:** 2020-12-05

**Authors:** Harpa Lind Kristjánsdóttir, Sigrún Sigurðardóttir, Anna María Pálsdóttir

**Affiliations:** 1 Vocational Rehabilitation Center of the Westfjords, 400 Ísafjörður, Iceland; 2 Faculty of Graduate Studies in Health Sciences, University of Akureyri, 600 Akureyri, Iceland; sigrunsig@unak.is; 3 Department of Work Science, Business Economics and Environmental Psychology, Swedish University of Agricultural Sciences, P.O. Box 88, SE-230 53 Alnarp, Sweden; anna.maria.palsdottir@slu.se

**Keywords:** nature-based rehabilitation, vocational rehabilitation, salutogenesis, restorative environment, health promotion, blue health, forestry, perceived sensory dimensions, landscape architecture

## Abstract

This study aimed to investigate if proposed restorative attributes according to attention restoration theory and supportive environment theory could be experienced and identified in Icelandic landscape and contribute to a restorative experience in nature sites in rural Iceland. A prospective mixed-method study was conducted over the period of one year. Seven different nature sites that were considered likely to have restorative qualities were selected for the evaluation i.e., three forest sites, three seashores, and one park in and in the vicinity of Ísafjörður, Iceland. Each site was evaluated regarding how the participants experienced its restorative qualities and how a stay therein affected their mental state. Nature visits were offered once a week, where the participants visited one of the seven locations for two hours. The findings show that the participants perceived and experienced nature sites as having the characteristics of a restorative environment and that staying at the nature sites positively affected their mental state. External conditions, like weather, which can affect nature visits, were rarely a hinderance. Thus, it can be concluded that numerous coastal areas, forests, and parks in Iceland, especially in rural areas, might possess restorative qualities as well. This result shows that wild and open nature in North West Iceland has the characteristics of a restorative environment and can be utilized for health promotion.

## 1. Introduction

There is growing evidence that both passive and active exposure to nature can have a positive impact on human health [1,2,3]. Research on the role of nature for human health has increased significantly [4]. The increasing prevalence of lifestyle diseases, which are related to changes in the industrial structure and people’s lifestyles, is thought to be one of the factors supporting an interest in understanding nature’s role on human health and well-being [5,6]. The methods of the biomedical model, which had prevailed until the end of the twentieth century, have been successful in the treatment of infectious diseases and many physical ailments. However, these are not equally useful in preventing diseases that appear as consequences of e.g., stressful and sedentary lifestyles [4]. Therefore, a broader view of disease treatment, prevention, and health promotion is called for [5]. The idea that factors other than the pathogens themselves, such as social conditions and the environment, influence disease development has become more intense [7]. People’s views also began to focus on factors that could contribute to their health, and the concept of salutogenesis emerged. The creator of this concept, Aaron Antonovsky [8], believed that instead of focusing on pathogens and risk factors, it is necessary to look more at the factors that could contribute to improved health and well-being, both in the individual and the environment. Nature is one of the factors that is considered to be salutogenic and can promote a healthier lifestyle and well-being [4,9]. Questions have been raised about the health-promoting qualities of the natural environment that can support human health [10,11]. According to the attention restoration theory (ART) [12,13], four main perceived qualities can be health promoting: being away, extent, fascination, and compatibility. Further, the theory is based on the idea that humans have two kinds of attention: directed attention and fascination. Directed attention requires a lot of effort of the mind, and too much work that requires directed attention can cause directed attention fatigue. Fascination, on the other hand, is considered capable of rebuilding and promotes the ability and endurance to concentrate, and the experience of nature is considered as being important to recover from directed attention fatigue [14]. The demand for directed attention has increased significantly in recent decades, e.g., with increased technology and information flow. At the same time, opportunities for rest during the day have diminished, resulting in people being less able to cope with the mental fatigue that arises [12,13]. Although the natural environment is not the only environment that can be considered health-promoting, the health-promoting properties of the natural environment seem to be extremely important. The reason for this is that it easily attracts people’s attention at the same time as it makes minimum demands on directed attention [15].

Supportive environment theory (SET) [16] is related to the development of man in a social, cultural, and physical environment, from the time when the natural environment was dominant and society and daily life were much simpler than they are today. It assumes that people need support from the environment to develop and maintain their health, both physically and mentally, to varying degrees, depending on the individual and the situation. SET defines individuals’ needs for support from the environment based on their executive functions, the need to look inward, and the ability to participate outward. Executive functions refer to people’s real ability to change their environment and create the environment they want to live in. Attention is focused on how to plan the environment so that the needs of all groups are met, and the executive functions are restored [16,17]. SET integrates several theories, where the eight perceived sensory dimensions (PSDs) are an important part. Other theories and concepts include: Antonovsky’s theory of “salutogenesis” and “sense of coherence”; affordance, a term coined by the psychologist James J. Gibson [18]; and the term “social quietness,” which is a relatively newly defined factor that characterizes a health-promoting environment [17,19]. According to SET, factors discussed in all of the abovementioned theories and concepts can contribute to the creation of a supportive environment and thus promote improved mental health and well-being and the restoration of executive functions. Grahn and Stigsdotter [20] propose eight PSDs: nature, serene, refuge, space, prospect, culture, rich in species, and social. Each of these dimensions indicate a generally perceived need for support from the environment [16,20]. Furthermore, people often seek an environment that they find supportive and safe, which are considered to be prerequisites for people to be able to let go, be themselves, absorb information, and recover [16,17,21,22].

Within the field of evidence-based health design in landscape architecture [23,24], the abovementioned theories have been applied in the design of healing gardens and supportive outdoor environments for individuals in nature-based rehabilitation. Recent examples of such implementation are two research behavioral settings i.e., Alnarp Rehabilitation Garden in Sweden and Nacadia in Denmark [24,25,26,27,28]. Both health gardens are the subjects of continuous research and, as such, a source of new knowledge and understanding of the restorative potential of being in a natural milieu. From this research of attributes, ART/PSD are proposed as having a restorative effect when in place in the environment.

In many countries, systematic use of nature in the treatment of various health problems has increased [29,30,31,32,33]. However, in Iceland, this has received little attention, but the results from a recent study suggested that nature walks in a woodland setting can have higher potential for stress reduction compared to gym or video settings with nature [34]. However, the study did not identify specific attributes or qualities in the outdoor setting that were perceived as restorative or inducing stress reduction. Therefore, this study attempts to identify certain qualities and attributes in the Icelandic nature that are perceived restorative.

The aim of this study was to investigate if proposed restorative attributes according to attention restoration theory and supportive environment theory could be experienced and identified in Icelandic landscape and if these could contribute to a restorative experience.

## 2. Materials and Methods

This study was conducted as a longitudinal prospective study, including both a quantitative and qualitative approach, examining individuals’ experiences and perceptions of seven different nature sites in North West Iceland. The study was carried out over a one-year period. It was a mixed methods approach using self-monitored questionnaires and a focus group interview for evaluating possible restorative potential and the attributes of Icelandic nature at seven different locations. The locations were a park, three forest sites, and three seashores (see Section 2.3 for descriptions of each site).

### 2.1. Data Collection

#### 2.1.1. Quantitative Outcome Measures

To evaluate the restorative potential of each of the locations, two different questionnaires were used to evaluate the participants’ (1) perceived mental state and (2) perception of the environment regarding the restorative qualities in each location, according to the ART theory. Both questionnaires were translated from English into Icelandic and then back-translated, according to an approved translation method [35] and tested by individuals unrelated to the study before it began.

##### A Questionnaire on Mental State

This short questionnaire was adopted from a study by Dolling et al. [36] and Sonntag-Öström et al. [37]. The questionnaire addressed: tension (tense/relaxed), fatigue (exhausted/alert), happiness (sad/happy), irritability (irritated/harmonious), restlessness (restless/peaceful), and clear-headedness (mentally divided/clear-headed). Each alternative was rated on a scale of 1 (not at all) to 10 (very much). A high number indicated a better state of mind. Each time the participants went out to a nature site, they filled out the questionnaire both before the visit and after they came back from the visit. Attempts were made to go to all of the locations at least once each season to cover seasonal changes and variations.

##### A Questionnaire on Perception of the Environment

The questionnaire was comprised of 12 questions [36] concerning restorative qualities that are considered to be characteristic of a supportive environment, according to ART [12,13]. These included: being away, extent, fascination, and compatibility. There were two questions for each of the three qualities: fascination (something captures my attention; it is secretive and mysterious); being away (I can just be; I release thoughts about routines), and compatibility (all fits together naturally; it is simple and undramatic), and there were three questions regarding space (I have an overview; I am a part of the whole; here is space). Furthermore, there was one question each about safety and light. Responses were on a scale of 1–7, where 1 meant “strongly disagree” and 7 meant “strongly agree.” All participants answered the questionnaire after each visit to a nature site.

#### 2.1.2. Qualitative Outcome Measures

##### Other Means of Data Collection—Photos, Videos, and Researchers Notes

Many photos and videos were taken throughout the research process, both by the researcher and the participants, even though they were not encouraged to take any. Instead, they were encouraged to just enjoy being present in the nature and not using their phones. However, they often felt the need to capture the beauty and grandeur they witnessed, and those pictures then became part of the research data. The researcher compiled the pictures and categorized them by place. They were then viewed in connection with focus group interviews with participants and used to support the data analysis.

Not only were photos and videos viewed, but notes the first author wrote throughout the process were reviewed, e.g., specifically finding comments that had been jotted down about the progress of the study, comments from participants on various things, and what they thought was positive or could be different. This information was used to support the analysis and the interpretation of the data.

##### Perceived Sensory Dimensions

After summarizing the results from the questionnaires, viewing photos and videos, and reading the notes, a focus group interview was conducted with the participants. The aim was to gain a better understanding of the group’s experience of participating in the nature visits and in the nature sites. A focus group interview was considered suitable for this purpose [38,39], where it was thus possible to hear the experiences of more participants than in individual interviews with e.g., two to three participants. This method also generates spontaneous discussion on topics not foreseen. The focus group interview took place in December 2019, and altogether six of 11 participants took part in the interview. Due to personal circumstances, the remaining five could not participate. The interview began by looking at the photos taken at the sites during the nature visits. Thereafter, they were asked about their experiences of staying in the nature sites, what they thought was positive, and what they thought was negative. Open-ended questions were posed as well as follow-up questions on subjects brought up by the participants.

### 2.2. Analyzing Data

#### 2.2.1. Quantitative Data

When analyzing the data, quantitative data from the questionnaires were recorded in a spreadsheet and the statistical program SPSS was used for all statistical processing.

##### A Questionnaire on the Perception of the Environment

The results of all of the questionnaires for each site were grouped together, the questions were categorized according to what qualities of the ART theory they described, and the average value from all of the answers concerning each ART characteristic was calculated. The average for all of the answers concerning light and safety for each location was also calculated. To see if there was a difference in experience by season, an ANOVA test was used to see if there was a difference in the average values, and a Bonferroni test was used to see if there was a difference by season.

##### A Questionnaire on Mental State

The results before and after a visit to a nature site were compared, and this was done for all of the questionnaires for all nature sites. To compare the participants’ mental states before and after their stay at the nature sites, a paired sample t-test was used, which compared the averages of the same group (e.g., nervous/relaxed before and after). The level of 0.05 was considered statistically significant.

Due to the relatively small number of responses for each site, a t-test was performed on all of the answers for a comparison of the participants’ mental states before and after a stay at a nature site, regardless of the location

#### 2.2.2. Qualitative Data

An audio recording of a focus group interview was listened to in its entirety and then listened to again, and all comments that included descriptions of participants’ experiences of the nature sites were written verbatim. That text was then scrutinized and analyzed. Then, participants’ comments and descriptions regarding their experiences of the environment and how they felt in the nature sites were coded according to the properties that characterized each of the eight PSDs: serene, nature, refuge, space, prospect, rich in species, culture, or social dimensions. As a result, it was possible to determine if the participants had experienced any PSDs and, if so, which ones. Each of these dimensions indicated a generally perceived need for support from the environment [16,20].

There are eight PSDs, and their names and main characteristics are:

- Nature: The fascinating impact of wild nature, which has developed on its own terms. An experience of the inherent force and power of nature.
- Serene: Serenity, peace, and sounds of nature (such as wind, water, or birds). No disturbances, safe and secure. A “holy” place.
- Refuge: Sheltered place. An enclosed, safe, and “secret” place, where you can relax and be yourself.
- Space: A spacious, cohesive whole that provides a restful feeling of “stepping into another world.”
- Prospect: Open space and vistas. A place that invites you to stay.
- Culture: Traces of human values, beliefs, structures, and historical heritage over time.
- Rich in species: There is a large variety of plant and animal species in the area.
- Social: A meeting place for festivity, pleasure, and entertainment. A venue for meetings and social gatherings.

One question (protected from visibility) was not used in the analysis of the data and the presentation of the results, as it was not relevant regarding the ART characteristics. Nevertheless, participants answered this question as a part of the questionnaire, which could be used for further analysis for another occasion.

### 2.3. The Study Locations

**Figure 1:**
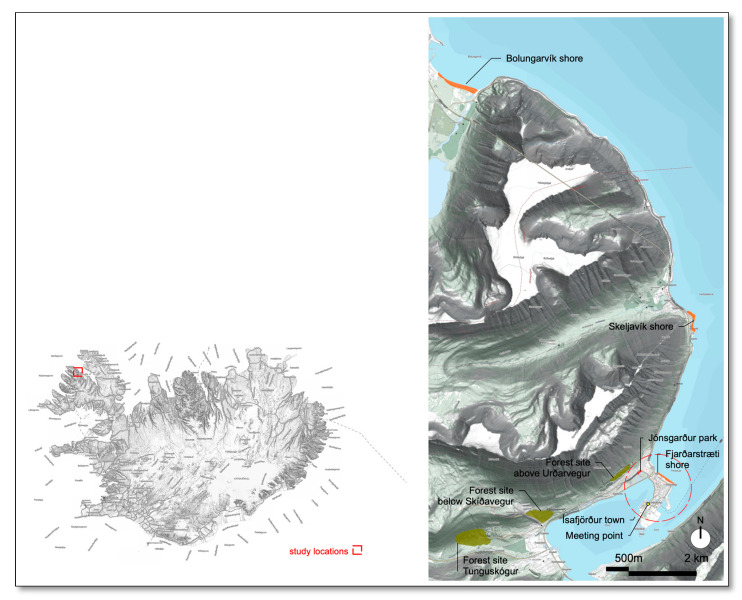
The map on the left shows where in Iceland the study took place. The map on the right shows the seven nature sites with seashores colored orange, forests green, and the park red.

The study was conducted in Ísafjörður, located in the Westfjords, which is the northwestern corner of Iceland. Seven different nature sites in and near the town were selected for the study. When choosing the nature sites, consideration was given to areas with vegetation, forest sites and parks, and coastal areas; the sites were both within and outside of the town. The qualities of the sites were not formally evaluated for the study, but it was considered positive to choose a different kind of site, as it offers more variety in the nature visits. Other aspects taken into consideration were that the nature sites were in the vicinity of or within a manageable distance from the town, and were easily accessible, including some that could be reached from the groups’ meeting point by foot, with distances ranging from 500 m to 14 km away (Figure 1). Jónsgarður Park, Fjarðarstræti Shore, and the forest site above Urðavegur could be reached by foot, but the other sites were accessed by cars. The nature sites should be large enough for hosting a group of participants as well as have possibilities for them to seek solitude, have the potential to be restorative, and have the potential to offer a restorative experience. Therefore, crowded places were excluded. According to research [17], the possibility to seek solitude is an important aspect of a restorative environment as well as being free from crowds and the demands of social interaction [20,23].

#### 2.3.1. Park

##### Site 1—Jónsgarður

**Figure 2:**
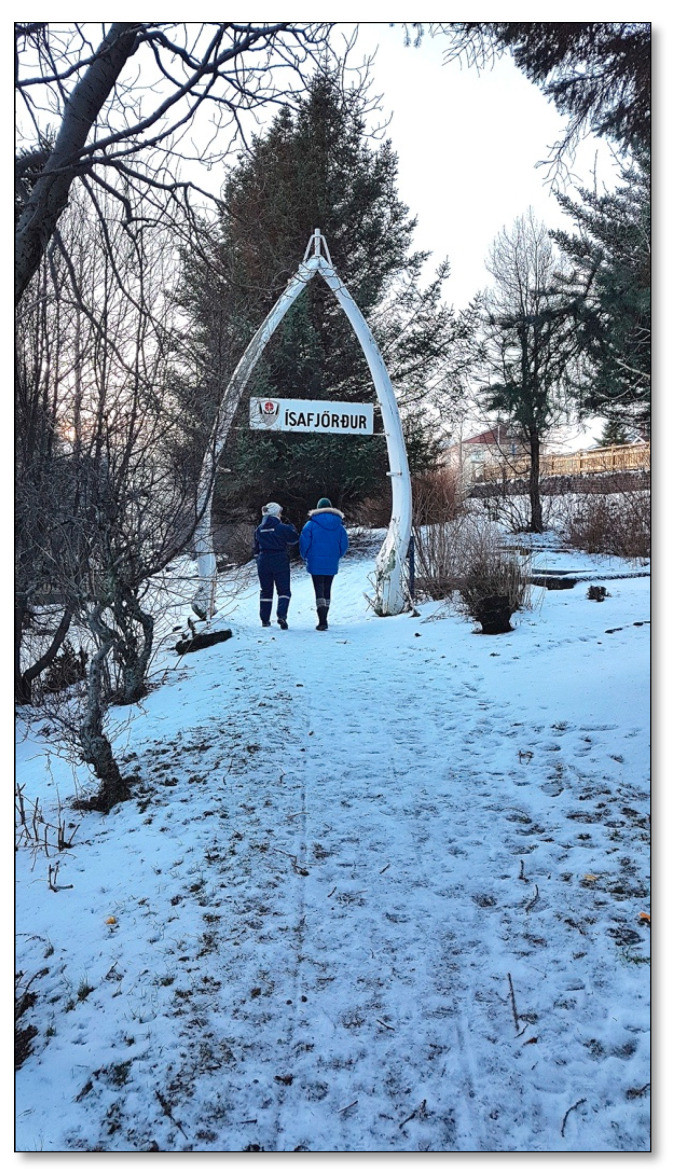
Jónsgarður Park.

A park located in Ísafjörður is about 4000 m^2^ in size (Figure 2). Trees were first planted in the garden in 1925, and the oldest trees are probably close to 100 years old [40]. Most of the trees are spruce, but there are also a lot of shrubs and terrestrial vegetation. The garden is grassy, except for paths that are paved with gravel, and there are raspberry and currant bushes. There is a small 15–20 m^2^ platform, intended for outdoor teaching of preschool and primary school children, with benches along its two sides. Elsewhere, there are logs lying around a fireplace. The park is often desolate, but there are often people walking there during the summer. Jónsgarður is one of the two parks in Ísafjörður considered as possible nature sites to study, as they are the only green spots in the old town (the area in the red circle on the map). However, the other park was deemed too busy for this purpose, as it is located beside the town center and the school.

#### 2.3.2. Coastal Areas

##### Site 2—Fjarðarstræti Shore

**Figure 3:**
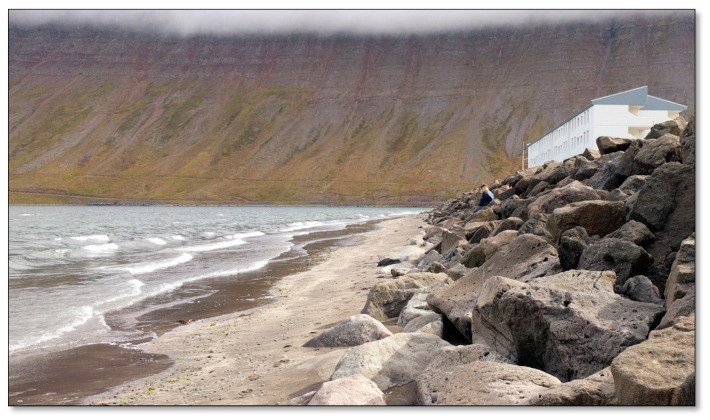
Fjarðarstræti Shore.

Fjarðarstræti Shore is about 450 m long, with black sand (Figure 3), which has formed below a loaded breakwater in recent years. Between the big rocks at the bottom of the breakwater, it is somewhat sheltered from view from the road, from other people on the shore, and also from harsh weather and winds. There is little flora and fauna on the shore, except for kelp, shells, occasional crucian carp, crab, and other things that have washed ashore. In addition, green beach vegetation can be seen along the shore in the spring and into the autumn. Similar to the above site, the shore is very often desolate, except for in the summer, when there are sometimes people walking and playing there, especially during nice weather. This site was chosen because it is the only spacious sand shore within a walking distance from the town, it’s never crowded, and there is a great ocean view.

##### Site 3—Bolungarvík Shore

**Figure 4:**
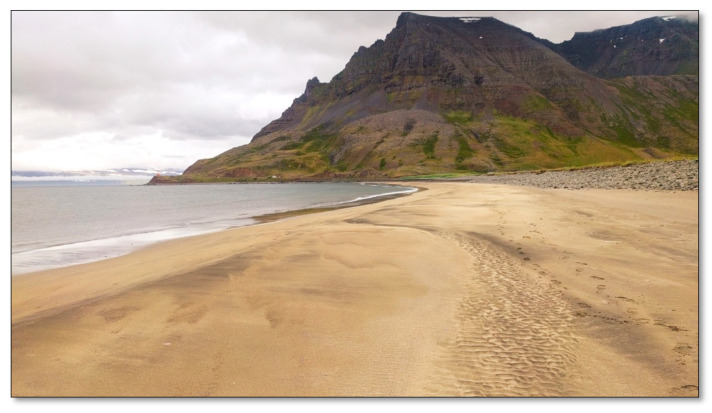
Bolungarvík Shore.

Bolungarvík Shore is about a one kilometer long, natural, undisturbed sandy shore (Figure 4). The beach is extensive, and a variety of patterns form in the sand when the waves recede. There is a great view, both of the mountains on either side and of the sea. From the beginning of summer until autumn, there is considerable coastal vegetation that forms a green ridge at the top of the shore. Stockfish hovels are found at an approximately 100–200 m section, almost completely down by the shore ridge. Very seldom can you see people walking around on weekdays. This place was chosen because of its spacious, unfrequented, undisturbed nature, with a great horizontal view.

##### Site 4—Skeljavík

**Figure 5:**
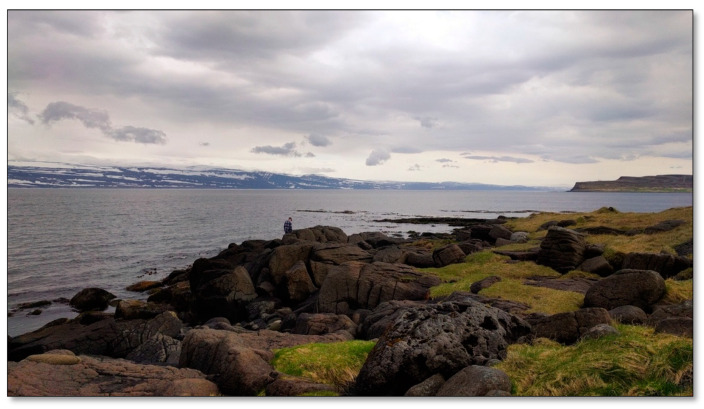
Skeljavík Shore.

Skeljavík is a 150 m sandy shore, just south of Hnífsdalur (Figure 5). At the end of the shore, there is a 200–300 m coastline where rocks and cliffs lead into the sea; above the cliffs is a grassy, hilly area. There are many ditches and depressions in the landscape, which make it easy to disappear from the sight of others. When the sea level is low, you can find a variety of sea insects, mussels, snails, and more that are left in small puddles along the shore. Lots of birds and seals can be seen. Various kinds of seaweed and kelp e.g., dulce, are found along the shore. There is also a lot of coastal vegetation, which blooms with many small flowers in early summer and stays in bloom until autumn. There is a great view of the ocean. The area is hardly used for outdoor activities and is almost always uninhabited. It is a hidden piece of undisturbed nature with a horizontal view, which is why it was chosen.

#### 2.3.3. Forests Sites

##### Site 5—Forest Site Below Skíðavegur

**Figure 6:**
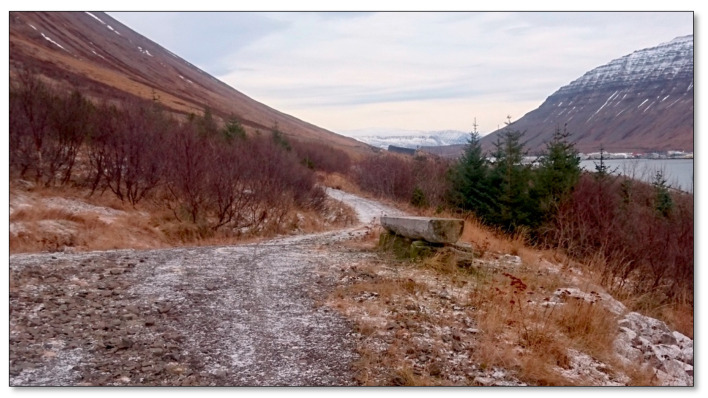
Forest site below Skíðavegur.

The forest site was planted around 1990 (Figure 6). It is part of a larger outdoor recreation area, created after the construction of avalanche defenses just west and above the area around the year 2000. A wide gravel path runs through the area with narrower paths leading from it. There is a lot of birch, willow, spruce, and pine, as well as large aspen trees that frame a small grove by a bridge over the river that flows through the area. By the bridge, there is a bench and a small lawn with good shelter. The place is connected to a larger network of footpaths, and there are few people walking there during working hours on weekdays.

##### Site 6—Forest Site Above Urðavegur

**Figure 7:**
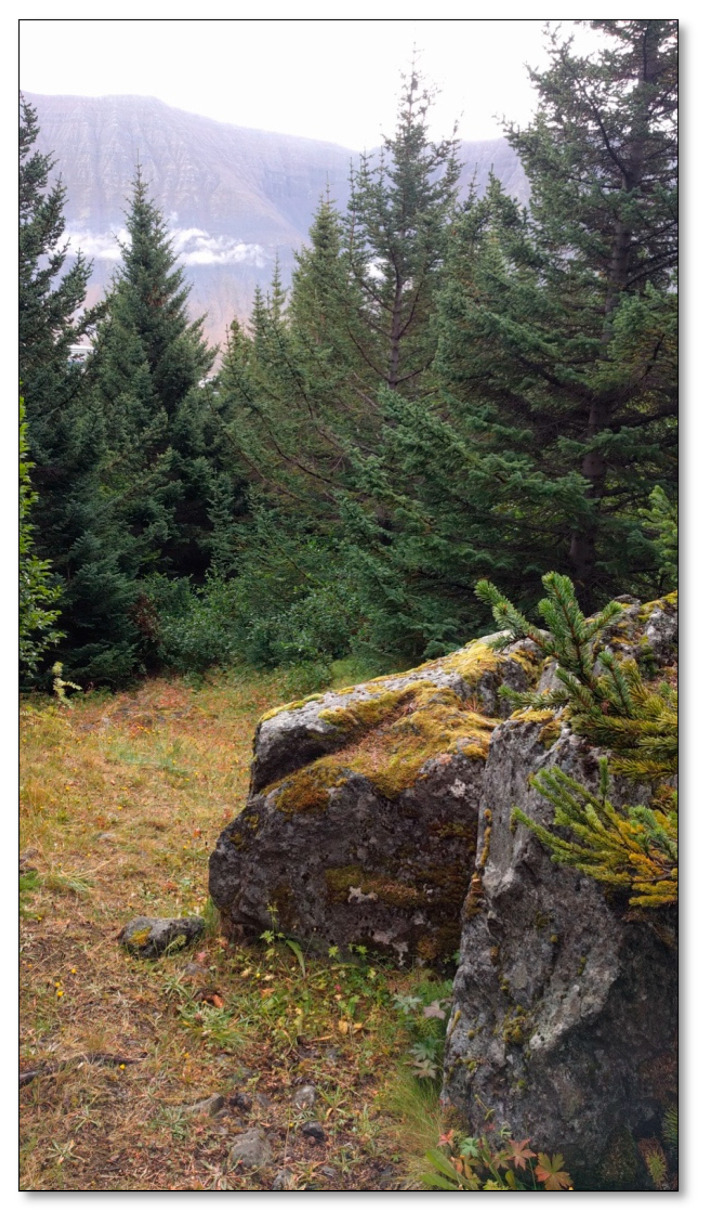
Forest site above Urðavegur.

The first trees were planted in the mature forest plot in 1945 (Figure 7). Most of the trees are conifers, but there are also some berry species, e.g., salmon berries, currants, and wild berries [41]. A gravel path runs through the forest across the hill, but narrower paths cross the area. Above the forest plot, there is a vegetation area, grass, and a variety of low vegetation, e.g., berry heather. Large rocks having collapsed from the slope have broken trees and formed sheltered spaces in the forest. It is mostly unoccupied on weekdays, though there are more people walking around in the summer than other seasons.

##### Site 7—Tunguskógur Forest

**Figure 8:**
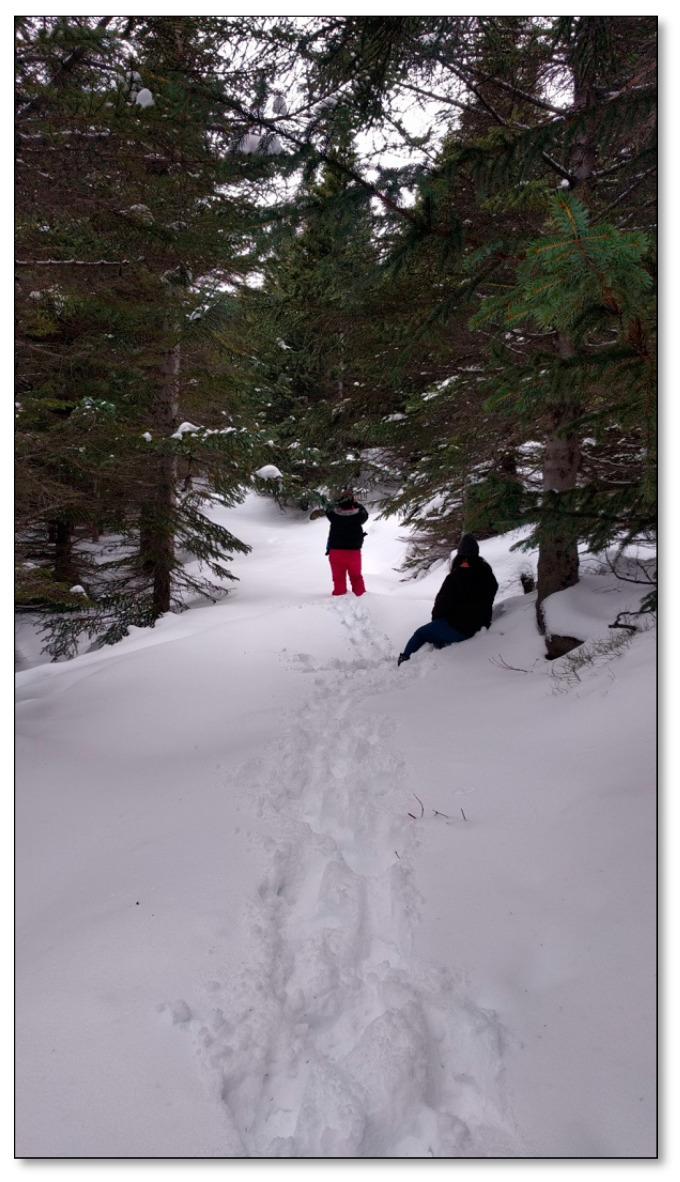
Tunguskógur forest.

Tungudalur is a valley quite sheltered from the wind and bad weather. There is a natural birch forest, with a forest plot (Figure 8) comprised of mainly conifers that were planted between 1955–1965 [42]. A gravel path runs through the coniferous forest; in addition, there are narrower paths around the area, and it is easy to access the forest through the many small clearings. Small wooden benches can be found scattered throughout, and by the main path, there is a table and some benches. The place is popular for outdoor activities, but mostly in the afternoons, on weekends, and in the summer.

Those three forest sites were considered as being most likely to possess restorative properties, as they are the most mature and largest forest sites in the area.

### 2.4. Participants

The participants were recruited from the Vocational Rehabilitation Center in Ísafjörður. They received both verbal and written information about the study and had possibilities to ask questions before deciding on participation. Participation was voluntary, and participants were free to withdraw anytime if they wished. A total of 11 individuals accepted the invitation, three men and eight women aged between 29–53, with an average age of 42. The period of participation varied from 4–11 months, with an average of 7.3 months.

### 2.5. Nature Visits

Nature visits were offered once a week, together with the first author. Each visit lasted for about two hours, including transport. The group met at a pre-determined meeting point before leaving together to go to the chosen site. Coveralls had been purchased that people could borrow when it was cold. Depending on the distance from the center, the group walked or drove to the nature sites. Upon arrival at the sites, the group walked around the area together; after a while, the participants were encouraged to go on their separate ways and be in solitude for up to 30 min. Participants were asked to find a place they preferred and sit there or walk around for a pre-determined time. People were encouraged to pay attention to the environment and sense it in every way possible: by listening to the sounds at the site, smelling, touching the ground/surface, and feeling the wind, cold, or rain, etc. After some time in solitude, the group gathered together again, talked a little about their experience of the site, looked at things they found special, and then went back.

### 2.6. Factors to Consider When Planning

There were many factors to be considered when planning nature visits, such as changing and extreme weather and seasons, and different circumstances at the sites as a result of those factors. Winter coveralls were bought, and participants could borrow them if they wished. From the beginning, participants were informed that the plan could change if circumstances so required e.g., due to weather.

### 2.7. Ethics of Research

Research must meet certain ethical requirements, and it is important that researchers fulfill them and maintain the trust of those participating [43]. An inquiry regarding ethical approval was sent to the National Bioethics Committee of Iceland; however, according to the committee, there was no need to apply for a permit to collect data for this study. Nonetheless, the study followed the recommendations of the WHO for conducting research including humans i.e., written and verbal information about the study, possibilities to ask questions before deciding on participating in the study, participation on a voluntary basis, and the participants could withdraw for any reason at any point, without any consequences. All data are presented anonymously and on a group level.

## 3. Results

In this chapter, quantitative results will first be presented i.e., the results from questionnaires on the perception of the environment and mental state. This is followed by the qualitative results on the perceived sensory dimensions.

### 3.1. Mental State

Pre and post measurements showed a significant difference in participants’ perceived mental state (Table 1). The most noticeable was their perceptions of being more relaxed and clearheaded after staying at the nature sites. However, the participants also felt more peaceful, happier, and more alert. The only factor that did not change between the ratings was whether the participants felt irritated or harmonious after the walks.

### 3.2. Perception of the Environment

The results of the questionnaire indicated how participants perceived the environment and the extent to which they reported the restorative experiences described in ART. This was an indication as to whether the nature sites met the requirements for a restorative environment. Table 2 presents an overview of the results for all of the locations.

The overall score from the attention restoration scale for all of the nature sites ranged from 5.3–6.0 on fascination; 4.9–6.5 on extent; 4.7–6.1 on being away; and 4.8–5.8 on compatibility. Scores on light ranged from 5.6–6.6 and scores on safety ranged from 5.0–6.5. All scores were on a seven-point scale. Participants perceived shores as the most restorative nature sites, as they scored the highest on the ART factors (being away, fascination, extent, and compatibility). Skeljavík Shore had the highest score on fascination, extent, and compatibility and the second highest on being away. The park Jónsgarður is perceived as the least restorative, with the lowest score on extent, being away, and compatibility, and the third lowest on fascination. Forest sites come in between, with Tunguskógur forest at the top, with the highest score on fascination (along with Skeljavík Shore), the third highest on being away, and second highest on compatibility. The same pattern was seen regarding perceived security and light on the nature sites. The results from the questionnaire did not show any significant differences in the participants’ experiences of characteristics of the nature sites between the various seasons.

### 3.3. Perceived Sensory Dimensions

Results from the focus group interviews indicated that the participants experienced all dimensions except the social dimension at the nature sites (Table 3). Descriptions of those experiences were analyzed and categorized based on the specific dimension they conformed to.

#### 3.3.1. Nature

All participants sensed this dimension at all of the nature sites. They experienced the inherent force and power of nature, both through the sound of the waves and the presence of the wind by the sea. In the forest site above Urðavegur, there are big rocks that have fallen down a mountain side and broken old trees; here, some new trees have started to grow from the stock plants. Participants found it “interesting to see how nature behaves when it is left alone,” and they found it fascinating how the trees “just keep on growing like nothing has happened.”

Participants experienced that the sea and the surfs “gave power”, and that the sound of the waves “breaks down negative thoughts and bad feelings.” Experiences of the magical effects of nature and how it has evolved on its own terms were also described by the participants, both at the shores and in the park and forest sites. They were fascinated by many of nature’s phenomena, e.g., rocks shaped by the sea, diversity and contrasts in the landscape, nuances, strange plants and trees, brooks, and waterfalls. They felt an energy and a calming effect from the trees and found it soothing just to sit and watch the trees, the sea, and nature in general; they often felt that all of their senses were activated. One called it an “awakening of the senses.”

#### 3.3.2. Serene

Stillness, peace, and security were experienced by the participants in all of the various nature sites. The sounds of nature, e.g., the noise from the sea, the sounds of birds, and the wind between the trees, were generally perceived as calming and soothing, like there was “some kind of peace between the trees.” The sounds were different when there was snow, as the snow dampened all of the sounds. The serenity was somewhat affected by man-made noises in the nature sites near the town or roads, as one could occasionally hear a car driving by or people talking. However, participants found that nature reduced the disturbance of the noise, like “nature and tranquility made the mind wander, even though there were cars driving down there.”

The participants liked to sit in silence and listen to the sounds from nature, and felt that it slowed down their thoughts and was “in fact somewhat like meditation.”

#### 3.3.3. Refuge

Experiencing a safe and closed sanctuary, where the participants could relax and just be, was characteristic of how the group described all of the nature sites. The sites were experienced as places where they could disconnect from their daily lives, relax, let go, and be themselves. One participant managed to “completely let go and wander around without keeping track of where I was going.”

Participants used small places at the sites, where they could disappear from the sight of others and be completely by themselves. Those were between big rocks in the breakwater, ditches and depressions in the landscape, and small clearings in the forests. Participants found solitude to be an important part of these trips. One of them said he felt very good in the forest “when I had found my place—I always went to the same place.”

#### 3.3.4. Space

This dimension was experienced in three of the seven nature sites. In Skeljavík Shore, the forest site above Urðavegur, and Tunguskógur Forest, the participants described having a feeling of calmness, like stepping into another world. “When I go into the forest, it is something completely different.” In Skeljavík Shore, the cliffs were considered interesting and fascinating, forming a kind of cohesive whole in the landscape. In the forest site above Urðavegur and in Tunguskógur Forest, the experiences of the forest were the same way, like a cohesive whole, another world where one could get away from everyday life, into the fascination of nature. “To me, it was just like stepping out of all the stress and into another world.”

#### 3.3.5. Prospect

This dimension was experienced in all three shores, Fjarðarstræti Shore, Bolungarvík Shore, and Skeljavík Shore, and in the forest site below Skíðavegur, which are all places with a view of the sea. All of the places are open with a beautiful view over a wide, open space, which was very much appreciated by the participants. They felt that looking at the view could “make you drift away”, and gave the participants a feeling of solitude and being in the present moment. Participants liked to have such a vast view and found it positive “to see far away, and not be cornered.”

#### 3.3.6. Rich in Species

Participants experienced this dimension in two nature sites, in Skeljavík Shore and Tunguskógur Forest. In Skeljavík Shore, the participants came across a variety of plant and animal species. They mentioned chirping birds and croaking ravens; they described holes in the ground that they thought were mink or fox holes, and there were footprints in the snow that they thought probably belonged to the inhabitants of one of the holes. Seals were also often seen in the bay. In Tunguskógur Forest, there were many species of plants in the area, which impressed the participants, as they always felt like they were seeing something new. They felt that they had learned to pay attention to nature in a different way than before and felt that they “saw much, much more details in nature than before.”

#### 3.3.7. Culture

In Skeljavík Shore, Jónsgarður Park, and Tunguskógur Forest, there were traces of human development and historical heritage. In Jónsgarður Park, there are jawbones from a large whale, which form a kind of a gate, which has been standing there for many decades. One participant expressed that of all the things the park had to offer, he was “most interested in the jawbones.” In Skeljavík Shore, a pallet and an old tire had apparently washed ashore in a surf, and reflected traces of economic development and industry in the area. In Tunguskógur Forest, there is a carving of a man with a beard in a tree stock, welcoming people as they enter the forest, and “makes one smile a little more.” There were also benches and tables, where the participants sat and rested on their walks.

#### 3.3.8. Social

Participants did not express any experience of the social dimension in the nature sites.

### 3.4. Weather, Seasons, and Safety

Participants were ready to go out in various weather and returned happy from walking and staying outside in cold weather, whether in snowfall or in rain. However, when strong winds were added to precipitation and/or cold, the experience of outdoor activities worsened greatly and suggestions for cancelling the nature visit increased, and on a few occasions, it was necessary to cancel the nature visits due to bad weather, or due to an extreme thaw, as it was not considered safe to take a group of people out for a walk on icy and slippery conditions. Under such conditions, the outdoor nature sessions were cancelled as a safety measure, as participants’ safety could not be compromised. The presence of snow had also affected access to places, and where the snow accumulation was high, it could be difficult to walk around. Local ice accumulation also had an impact on accessibility. As previously stated, there were no significant differences in participants’ experiences of the characteristics of the nature sites between the various seasons.

On a few occasions, the group went to the shores when the tide was high. Although it was possible to walk on the shores in most cases, there was less space available than when the tide was low. Participants then felt a little cornered, and some found it uncomfortable to not be able to choose how far they were from the water. Once, the group chose to walk above the shoreline, when the sea seemed uncomfortably close when they went down on the shore.

### 3.5. Summary of the Results

There was a significant difference in all aspects of participants’ mental states, except irritability, before and after staying at the nature sites. All of the nature sites were experienced as having the characteristics of a restorative environment. The four restorative experiences of ART and the PSD dimensions, nature, serene, and refuge, were experienced in all sites. The PSD space was experienced in three sites, namely in the two mature forest sites (forest site above Urðavegur or Tunguskógur Forest) and the shore with a large grassy area with cliffs (Skeljavík Shore). In Skeljavík Shore, the dimension of culture was also perceived, as it was in the park and in Tunguskógur Forest. The dimension of prospect was experienced in all of the shores, and the young forest site (forest site below Skíðavegur), and the dimension of rich in species was found at the shore with the grassy area and in an old forest site. Coastal areas were perceived as slightly more restorative than forest sites, and the park in the town was perceived as the least restorative.

## 4. Discussion

The findings of this study confirm that open, wild nature on the latitudes of the arctic circle can be perceived as restorative year-round. The raw, powerful, and often imposing characteristics of nature in the Westfjords of Iceland reflect the properties of a restorative environment, according to both attention restoration theory and supportive environment theory. The area also offers the characteristics of supportive environments and can improve people’s mental state.

### 4.1. Mental State

Staying in the nature sites had a significant positive effect on the participants’ mental state each time they went out to a nature site; however, it is not possible to say whether that effect lasted over a longer period of time. This is in line with the results of two Swedish studies [36,37], except for the harmonious scale, which did not change at all during the nature sessions in this study. On the other hand, results of another study have shown that people’s “stress levels were reduced to a significantly greater extent than feelings of being well-balanced were enhanced” [44]. This could also be explained by the fact that social interaction is demanding for participants and that the presence of others interferes with their connection to nature and experience of social quietness [19]. SET also describes the relationship between executive functions, participation, and individuals’ needs for support from the environment [17]. It is also possible that the time people spent in nature sites was not long enough, and that a longer time would have had a positive effect on mental balance [13,44]. In this study, participants spent up to 30 min in solitude at the nature sites versus two hours, as in the study by Sonntag-Öström et al. [37]. In both studies, the time in solitude was a highly appreciated part of the nature sessions that could have fulfilled participants’ need for social quietness [17]. Interaction between persons in the group could also have influenced the ratings on the harmonious scale.

### 4.2. Perception of the Environment

When living in an area with little vegetation and forests but an endless coastline, the results of this study are particularly interesting. They show that the coastal areas evaluated in this study have fairly higher restorative properties than the other sites in the study. Their common characteristics are wide open spaces, brightness, and a magnificent view. Besides the everchanging, mesmerizing sea surface and sounds that can range from low and soothing to rumbling, the coastal areas can easily be experienced as a world of their own. This is reflected in the fact that extent is the highest rated quality for the coastal areas. These coastal sites also have three of the four highest scores on fascination, two highest on being away, and the highest score on compatibility. This is in line with the study by Sonntag-Öström et al. [37], where participants considered proximity to water as very beneficial; furthermore, they preferred the forest setting by the lake the most because of the water, the light, and the openness. Another study [45] similarly showed the advantage that areas by water have in terms of restorative properties. It has been shown that a sensory input from nature, such as birdsong and sounds from the sea, can evoke physical responses that are important for human health and well-being. It is possible that those sounds and people’s reactions to them contribute to the health-promoting effect of staying in coastal areas, where the sea level is high [46]. Given the fact that Iceland has approximately 6000 km of coastline [47], there is a great restorative potential, even though not all areas can easily be accessed and utilized for nature visits. Out of Iceland’s total surface (103,492 km^2^), only about 3200 km^2^ are forest, vegetation cover, and agricultural landscape. The majority of the research that has been done on the effects of nature on people’s health and well-being have focused on urban vegetation areas and forests. Areas along the shores of lakes and seas have been less studied, but attention has started to shift toward them [48]. Specifically, studies have shown positive effects of proximity to coastal areas on health and well-being [48,49,50,51,52].

The ART characteristic that was most often mentioned by the participants was the fascination of nature itself, e.g., the power and sound of the waves, beautiful views, the admiration of a small waterfall, and a strange pine tree. The soothing presence of old trees and varied cliffs fascinated participants, both in terms of appearance and texture. Those are the same aspects of nature that have also fascinated participants in other studies [37,53]. Research show a correlation between how a natural environment is experienced and its health-promoting properties [28,54,55]. Unspoiled nature is also considered to be the type of environment that best holds the properties that are considered restorative according to ART, with fascination being a central component in the restorative experience [13]. The Tunguskógur Forest, along with the Skeljavík Shore, is the site where the participants experienced the highest fascination. It is the largest of the forest plots and located farthest away from the town in a sheltered valley. The forest captured the participants’ attention effortlessly, making them feel that everything changed when they stepped into this area and noticed the varied flora which they had never paid attention to before. This is exactly what makes an environment health-promoting according to ART, i.e., when the environment gently attracts people’s involuntary attention at the same time as it makes limited demands on direct attention [15].

### 4.3. Perceived Sensory Dimensions

In the focus group interview, the participants talked about their experiences at the nature sites. From their descriptions, the PSDs they experienced at each site were identified. Nature, serene, and refuge were the three dimensions experienced in all of the nature sites. As stated above, the presence of nature itself was the property most often mentioned and described by the participants. Thus, it is in accordance with the main characteristics of the PSD nature: “fascinating impact of wild nature” and “an experience of the inherent force and power of nature” [16,20].

Serene is one of the PSDs that is considered important for restoration, as solitude and anonymity are very important aspects [23]. Although the nature sites evaluated in this study were open areas and not fenced, it was very rare that participants encountered other people around during the nature sessions.

The characteristics of PSD serene are: “serenity, peace and sounds of nature, such as wind, water, birds, no disturbances, safe and secure” [16,20]. This sounds just like a description of the nature sites, thoroughly described by the participants. Even in Jónsgarður Park, most of those properties apply, although to a fairly lesser extent than in the other sites. In some of the nature sites, participants could hear sounds from cars driving nearby; however, they did not find it disturbing, as they felt that the nature dampened the noise. The reason for this so-called sound screening could be that sounds of water have the ability to reduce unwanted noise and are perceived as being more restorative than technical sounds [56].

Refuge is the last PSD that participants experienced in all of the sites; it is also the dimension that studies have shown as being one of the most important restorative characteristics of the environment [20,23]. The characteristics of refuge, “sheltered place, an enclosed, safe and ‘secret’ place, where you can relax and be yourself,” have appeared in many different ways in the descriptions provided by the participants. Big rocks, high grass, hollows, trees, and bushes form enclosed and sheltered places in all of the nature sites; moreover, as they are rather infrequently visited, participants felt safe there and could relax. In most cases, there was enough space with the possibility to have an overview of the surroundings as well as an opportunity to get away if somebody approached, which is an important property of the refuge dimension i.e., not feeling trapped in a place [23].

The PSD prospect, which is characterized by “open space and vistas; a place that invites you to stay,” was experienced in four of the nature sites: the three shores and the youngest forest site. Factors that allow for the experience of the prospect dimension on the shores are quite obvious, as the most fabulous views as well as enclosed places as described above are found there. Some places in the young forest plot have a great view, where you can see between the trees over the fjord, as the trees are not so mature yet. The dimension space was also experienced in the two oldest forest sites and the Skeljavík Shore. The space dimension is described as “a spacious, cohesive whole that provides a restful feeling of ‘stepping into another world.’” Skeljavík is the only coastal area with quite a big grassy, hilly area, and cliffs that jet out into the sea. These sites gave the participants the feeling of wholeness and of being in a world of their own. Those five PSDs, nature, serene, refuge, prospect, and space, have been shown to be the most important dimensions of a supportive environment [19,23]. In this study, participants have experienced four of those five most restorative dimensions on all of the sites evaluated, except for the park Jónsgarður, where the dimensions were just nature, refuge, serene, and culture. Only two sites, the Skeljavík Shore and the Tunguskógur Forest, were rich in species. Those were the places highest on the ART characteristic fascination, and, in combination with PSD rich in species, seem to enhance the restorative qualities of the environment [23,57]. Thus, it may be concluded that all of the nature sites evaluated here have restorative characteristics according to the PSDs.

### 4.4. Weather, Seasons, and Safety

In the Westfjords of Iceland, weather is an unpredictable phenomenon, and can change suddenly. One of the things pondered on when planning the study was whether it was possible to practice nature visits in the Westfjords year-round. The results show that it is possible to do that, if there is a leeway and a will to some flexibility in the planning and to make changes when the external conditions require. As reported above, the participants took on the challenge of going out in a variety of weather conditions. Often, they were amazed at how refreshing it can be to go out for a walk in the rain or snow, and then, warm clothing was the key to a good experience. Sometimes, the weather affected the length of the stay at the nature sites and time spent in solitude, as it could get too cold to sit still. If the weather gets too bad, e.g., with sleet or cold and wind together, it can create negative exertion and stress instead of positive experiences. If a decision was made to not go out, it was always with that in mind. Flexibility is very important when planning nature visits in such circumstances. Participants were informed that plans could change due to the weather or outdoor conditions, and they received a message beforehand when that happened. Sometimes, it was also necessary to change the planned destination, as accumulation of snow or ice made it difficult to access some of the nature sites. When there were icy and slippery conditions outside, the nature visits could also be cancelled due to the safety of the participants, as that could not be compromised.

When planning walks on shores, there are some advantages to look at the tide table beforehand and choose to go there in low tide. Although it is possible in most cases to walk on the shores in high tides, there is less space available; this made participants feel more cornered, and some found it uncomfortable not being able to choose how far they could be from the water.

### 4.5. Methodological Considerations and Limitations of the Study

The study used both qualitative and quantitative research methods, which were well-suited for the subject of the study. The number of participants was small; therefore, it is not possible to generalize from the results of the study, either about the places examined or other similar places. The analysis of the participants’ experience of PSDs is based on only one focus group interview and, therefore, does not involve the same amount and depth of information as if it were a phenomenological study. However, the results provide a good indication of the characteristics of the natural sites that were part of this study.

## 5. Conclusions

The findings show that the different types of nature sites evaluated in this study had characteristics of a restorative environment according to attention restoration theory and supportive environment theory. The results show that staying at the nature sites had a positive effect on the participants’ mental states. This indicates that numerous coastal areas, forests, and parks in Iceland, especially in rural areas, may also be perceived as restorative. It is interesting that areas along the coast were perceived as having slightly more restorative properties than the vegetation areas. Thus, it can be inferred that the restorative properties of the coastal areas are at least the same as those of the vegetation areas, which increases the possibility of utilizing those areas, where there is little vegetation but plenty of beautiful untouched coastal areas, in a health-promoting purpose. External conditions like weather and snow accumulation are factors to be kept in mind when planning visits to nature sites. The results from the current study not only contribute to the knowledge of restorative landscape, but also point out the restorative value of the seashore which, in turn, undoubtedly increases the possibilities of utilizing the restorative potential of Icelandic nature for health promotion.

## Figures and Tables

**Table 1:** Change in mental state before and after staying at the nature sites.

				95% Confid. Interval of the Difference				
Mental State	Mean	Std. Deviation	Std. Error Mean	Lower	Upper	t	df	Sig. (2-Tailed)
Relaxed/tense	−1.270	2.434	0.283	−1.834	−0.706	−4.489	73	0.000
Exhausted/alert	−0.743	2.602	0.302	−1.346	−0.140	−2.457	73	0.016
Sad/happy	−0.635	2.168	0.252	−1.137	−0.133	−2.520	73	0.014
Irritated/harmonious	0.095	2.660	0.309	−0.522	0.711	0.306	73	0.760
Restless/peaceful	−0.811	2.262	0.263	−1.335	−0.287	−3.084	73	0.003
Mentally div/clear-headed	−1.014	2.296	0.267	−1.546	−0.481	−3.797	73	0.000

The mean shows the average difference between the two measurements before and after the stay at the nature sites. A minus in front of the numbers indicates positive changes in mental state, i.e., more relaxed, happy, peaceful, etc.; 0.05 indicates a statistical significance of changes between measurements (pre and post).

**Table 2:** Results from the questionnaire on the perception of the environment.

Nature sites	Fascination	Extent	Being Away	Compatibility	Light	Safety
Park						
Jónsgarður (n = 10)	**5.4**	**4.9**	**4.7**	**4.8**	**5.6**	**5.0**
Sea shores						
Fjarðastræti (n = 13)	5.5	6.1	5.4	5.2	6.4	6.1
Bolungarvík (n = 8)	5.7	6.4	6.1	4.8	6.5	6.3
Skeljavík (n = 8)	6.0	6.5	5.6	5.8	6.6	6.5
Average–shores	**5.7**	**6.3**	**5.7**	**5.3**	**6.5**	**6.3**
Forest sites						
Skíðavegur (n = 13)	5.3	5.6	5.6	5.5	6.2	5.2
Urðavegur (n = 7)	5.0	5.5	4.8	4.9	6.4	5.2
Tunguskógur (n = 17)	6.0	5.3	5.5	5.5	6.0	6.3
Average–forests	**5.4**	**5.5**	**5.3**	**5.3**	**6.2**	**5.5**

The responses are given on a scale of 1–7, where 1 = “strongly disagree” and 7 = “strongly agree.” The numbers are average values from all of the responses concerning each ART characteristic for each location, as well as questions about brightness and safety. The number of observations is given in the brackets. Bold numbers are average for each type of locations

**Table 3:** Overview of PSDs that participants experienced at the nature sites.

Nature Sites	Nature	Serene	Refuge	Space	Prospect	Rich in sp.	Culture	Social
Park Jónsgarður	X	X	X				X	
Sea shore Fjarðastæti	X	X	X		X			
Bolungarvík	X	X	X		X			
Skeljavík	X	X	X	X	X	X	X	
Forest Skíðavegur	X	X	X		X			
Urðavegur	X	X	X	X				
Tunguskógur	X	X	X	X		X	X	

X indicates that the specific PSDs were perceived and expressed by the participants.
